# Guidance for the Conduct and Reporting of Clinical Trials of Breast Milk Substitutes

**DOI:** 10.1001/jamapediatrics.2020.0578

**Published:** 2020-05-11

**Authors:** Katharine Jarrold, Bartosz Helfer, Mona Eskander, Helen Crawley, Jillian Trabulsi, Laura E. Caulfield, Gillian Duffy, Vanessa Garcia-Larsen, Deborah Hayward, Matthew Hyde, Suzan Jeffries, Mikael Knip, Jo Leonardi-Bee, Elizabeth Loder, Caroline J. Lodge, Adrian J. Lowe, William McGuire, David Osborn, Hildegard Przyrembel, Mary J. Renfrew, Paula Trumbo, John Warner, Barbara Schneeman, Robert J. Boyle

**Affiliations:** 1 National Heart and Lung Institute, Imperial College London, London, United Kingdom; 2 Bureau of Nutritional Sciences, Food Directorate, Health Canada, Ottawa, Ontario, Canada; 3 First Steps Nutrition Trust, London, United Kingdom; 4 Scientific and Technical Advisory Group on the Inappropriate Promotion of Foods for Infants and Young Children, World Health Organization, Geneva, Switzerland; 5 Department of Behavioral Health and Nutrition, University of Delaware, Newark; 6 Center for Human Nutrition, The Johns Hopkins Bloomberg School of Public Health, Baltimore, Maryland; 7 Department of Public Health Nutrition Standards, Food Standards Australia New Zealand, Canberra, Australia; 8 Section of Neonatal Medicine, Imperial College London, London, United Kingdom; 9 International Board of Certified Lactation Consultant Examiners, Fairfax, Virginia; 10 Children’s Hospital, Helsinki University Hospital, University of Helsinki, Helsinki, Finland; 11 Research Program for Clinical and Molecular Metabolism, Faculty of Medicine, University of Helsinki, Helsinki, Finland; 12 Medical Statistics, University of Nottingham, Nottingham, United Kingdom; 13 Research, *British Medical Journal*, London, United Kingdom; 14 Department of Neurology, Harvard Medical School, Cambridge, Massachusetts; 15 Allergy and Lung Health Unit, Melbourne School of Population and Global Health, University of Melbourne, Melbourne, Victoria, Australia; 16 Centre for Reviews and Dissemination, University of York, York, United Kingdom; 17 Division of Obstetrics, Gynaecology and Neonatology, University of Sydney, Sydney, New South Wales, Australia; 18 Department of Food Safety, Federal Institute for Risk Assessment, Berlin, Germany; 19 Mother and Infant Research Unit, University of Dundee School of Nursing and Health Sciences, Dundee, United Kingdom; 20 Nutrition Programs, Food and Drug Administration, Silver Spring, Maryland; 21 Department of Nutrition, University of California, Davis, Davis; 22 Centre of Evidence-Based Dermatology, University of Nottingham, Nottingham, United Kingdom

## Abstract

**Importance:**

Breast milk substitutes (BMS) are important nutritional products evaluated in clinical trials. Concerns have been raised about the risk of bias in BMS trials, the reliability of claims that arise from such trials, and the potential for BMS trials to undermine breastfeeding in trial participants. Existing clinical trial guidance does not fully address issues specific to BMS trials.

**Objectives:**

To establish new methodological criteria to guide the design, conduct, analysis, and reporting of BMS trials and to support clinical trialists designing and undertaking BMS trials, editors and peer reviewers assessing trial reports for publication, and regulators evaluating the safety, nutritional adequacy, and efficacy of BMS products.

**Design, Setting, and Participants:**

A modified Delphi method was conducted, involving 3 rounds of anonymous questionnaires and a face-to-face consensus meeting between January 1 and October 24, 2018. Participants were 23 experts in BMS trials, BMS regulation, trial methods, breastfeeding support, infant feeding research, and medical publishing, and were affiliated with institutions across Europe, North America, and Australasia. Guidance development was supported by an industry consultation, analysis of methodological issues in a sample of published BMS trials, and consultations with BMS trial participants and a research ethics committee.

**Results:**

An initial 73 criteria, derived from the literature, were sent to the experts. The final consensus guidance contains 54 essential criteria and 4 recommended criteria. An 18-point checklist summarizes the criteria that are specific to BMS trials. Key themes emphasized in the guidance are research integrity and transparency of reporting, supporting breastfeeding in trial participants, accurate description of trial interventions, and use of valid and meaningful outcome measures.

**Conclusions and Relevance:**

Implementation of this guidance should enhance the quality and validity of BMS trials, protect BMS trial participants, and better inform the infant nutrition community about BMS products.

## Key Points

**Question:**

What is the best way to ensure the validity of clinical trials of breast milk substitutes while protecting trial participants?

**Findings:**

Through a Delphi consensus project, guidance was developed to address issues specific to trials of breast milk substitutes assessing growth and tolerance, as well as trials of breast milk substitutes with other objectives. This consensus guidance summarizes best practice for the design, conduct, analysis, and reporting of trials of breast milk substitutes.

**Meaning:**

Use of this guidance, in conjunction with existing clinical trial regulations, should enhance the quality and validity of trials of breast milk substitutes, protect trial participants, and support the evidence base for infant nutrition recommendations.

## Introduction

Breast milk substitutes (BMS) are important nutritional products for infants who are not receiving breast milk. Most North American and European infants are exposed to BMS during their first year.^1^ Infants are sensitive to health effects of BMS owing to their early stage of development when consuming it and their potentially high level of BMS exposure when BMS are used as a sole source of nutrition. The potential association of BMS with population health is therefore greater than for many other nutritional products, and BMS need a scientifically robust evidence base so that caregivers and health care professionals can make informed feeding choices.^2,3^ Clinical trials that test BMS safety and evaluate changes in BMS composition or formulation are the foundation of this evidence base. Several groups have questioned the methodological quality of published BMS trials and, in turn, the robustness of their conclusions.^4,5,6,7,8,9^ Specific issues identified include risk of bias related to trial methods, lack of independence from BMS manufacturers, and less stringent regulatory oversight compared with drug trials.^6,10,11,12,13^ In BMS trials in which some infants are breastfed at enrollment, trials may also be failing to support the establishment and maintenance of breastfeeding in participants.^6,14^ These concerns, and the specific issues related to designing BMS trials that answer relevant scientific questions without undermining breastfeeding, suggest a need for new guidance for BMS trials.

We undertook a Delphi consensus to develop new standards for BMS trials. The new standards aim to support trialists in designing, conducting, analyzing, and reporting trials, as well as support regulators, critical appraisers, and reviewers in evaluating BMS trial reports. The guidance relates to intervention trials of BMS in infants enrolled prior to their first birthday, designed to demonstrate adequate growth and tolerance or other objectives. It is designed to complement other guidance such as that published by the US Food and Drug Administration or the European Food Standards Agency, Good Clinical Practice, or Consolidated Standards of Reporting Trials (CONSORT). Further details are summarized in the eAppendix in the Supplement.

## Methods

A 3-step Delphi consensus process was used to derive new methodological guidance for BMS trials. This Delphi consensus was undertaken between January 1 and September 30, 2018, with a consensus meeting on October 24, 2018. This method enables aggregation of the anonymous and independent opinions of an expert panel to reach consensus on agreed criteria.^15,16^ It is a systematic process of sequential rounds used to resolve clinical problems for which evidence is limited and the opinion of stakeholders is important but might be conflicting.^17,18^ We invited experts in BMS trials designed to demonstrate adequate growth and tolerance, BMS trials with other objectives such as supporting health and nutrition claims, BMS regulation, trial methods, breastfeeding support, infant feeding research, and medical publishing. Experts were identified through literature review and consultation with others working in these fields. Initial criteria were developed through review of existing clinical trial and BMS guidance, regulatory standards, and critical appraisals. We conducted 3 rounds of email questionnaires to generate, score, and refine criteria (Figure) and used published requirements for consensus^19^ (Table 1).^20^ The UK Health Research Authority was consulted and confirmed that this study did not require approval by a research ethics committee because it was not considered to be research on patients. Informed consent was obtained by email from all study participants. The protocols for this Delphi process and an associated systematic review are registered on PROSPERO (CRD42018091928).^21^ See the eAppendix in the Supplement for further details.

**Figure.**
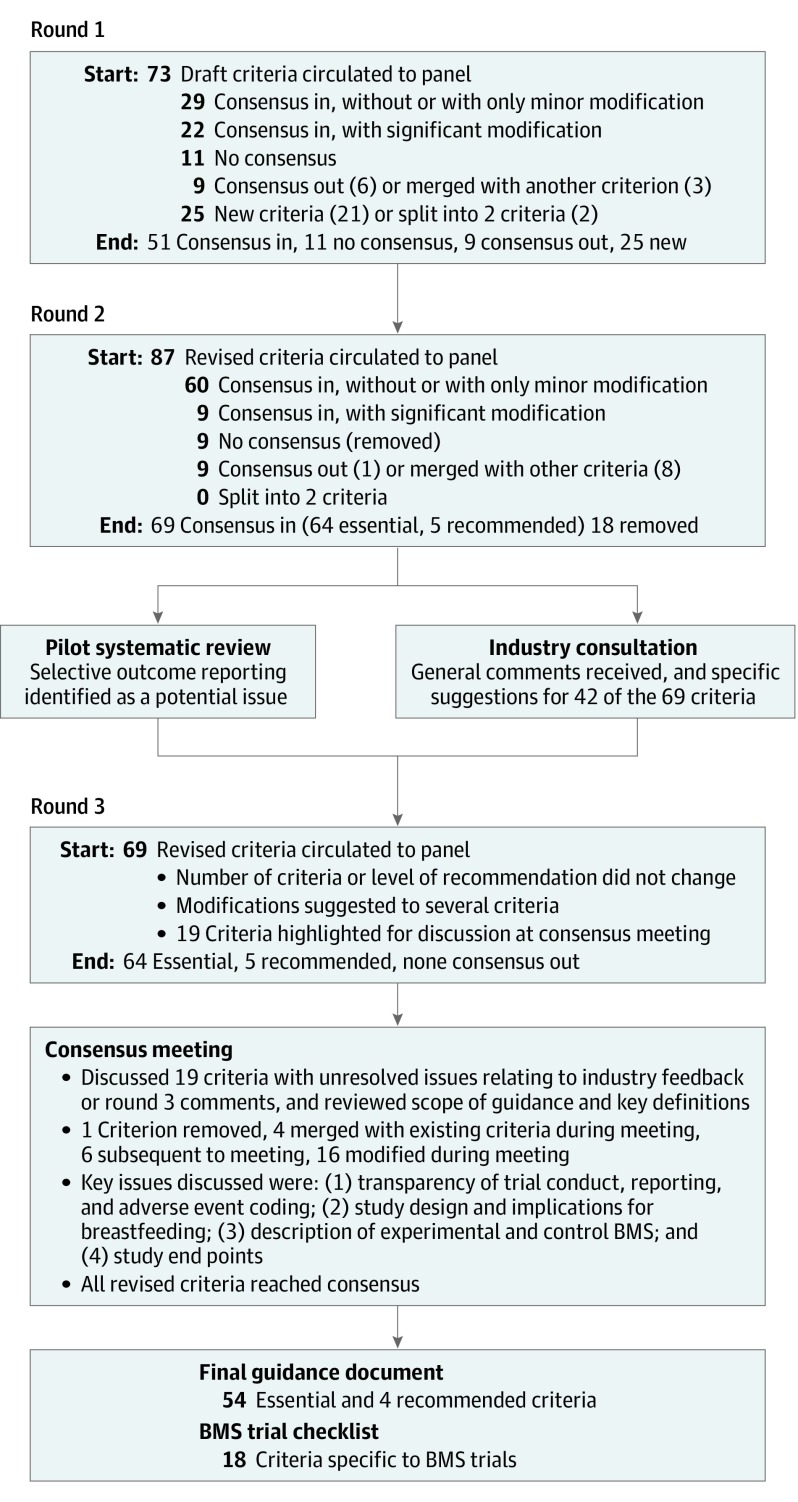
Summary of Delphi Consensus Process A summary of the actions taken during each step of the Delphi consensus process is shown. BMS indicates breast milk substitute.

**Table 1.** Definition of Consensus for the Delphi Process

Consensus classification^a^	Description	Definition
Essential	Consensus that the criterion is essential to the design or conduct of BMS trials	≥70% Of experts scoring as 7-9 and <15% of experts scoring as 1-3
Recommended	Consensus that the criterion is recommended with regard to the design or conduct of BMS trials	≥70% Of experts scoring as 4-6 and <15% of experts scoring as 1-3
Out	Consensus that the criterion should not be included in the core methodological criteria	≥70% Of experts scoring as 1-3 and <15% of experts scoring as 7-9
No consensus	Uncertainty about importance of the criterion	Anything else

Abbreviations: BMS, breast milk substitute; GRADE, Grading of Recommendations, Assessment, Development and Evaluations.

^a^ Consensus classification used the GRADE method. A score of 1 to 3 corresponds to not important, a score of 4 to 6 to corresponds to important but not critical, and a score of 7 to 9 corresponds to critical.^20^

Each round of the Delphi survey was piloted by the study team prior to initiation, and experts were given 3 to 4 weeks to complete each round, with regular prompts to maximize participation. The study team (K.J., B.H., and R.J.B.) was not part of the Delphi process and did not vote on the criteria.

### Delphi Round 1

Experts were asked to rate the importance of criteria that formed the initial guidance, using the GRADE (Grading of Recommendations, Assessment, Development and Evaluations) scale: a score of 1 to 3 corresponds to “not important,” 4 to 6 to “important but not critical,” and 7 to 9 to “critical.”^20^ If experts thought they could not comment on a criterion, they selected “unable to score.” Experts were also invited to provide free text comments, suggest adjustments to the wording of criteria, or suggest new criteria, and to comment on the scope of the guidance. The study team (K.J. and R.J.B.) summarized scores, anonymized comments, and classified criteria as essential, recommended, consensus out, or no consensus, as described in Table 1.^20^ All criteria other than consensus out were carried forward to round 2, together with proposed new criteria, proposed edits to existing wording, and any proposed merging or splitting of criteria. All changes or new criteria were highlighted in round 2, together with the anonymous comments from round 1.

### Delphi Round 2

Experts were asked to rate the importance of the revised criteria. For criteria repeated from round 1, experts were shown the consensus outcome and their own scoring. Experts were asked to reevaluate the criteria in light of the consensus outcome and propose further edits or comments, but could not add new criteria at this stage. Responses were classified and criteria modified in the same way as for round 1, but criteria that were still classified as no consensus were removed after round 2.

### Pilot Systematic Review

A pilot systematic review of a sample of recent BMS trials was undertaken by the study team (K.J., B.H., and R.J.B.) to evaluate adherence to the preliminary criteria generated in round 2. The findings were summarized for experts before round 3.

### Industry Consultation

Revised guidance after round 2 was sent to BMS industry representatives for comment on the feasibility and relevance of the proposed criteria. Breast milk substitute industry representatives were not invited to score criteria, but their feedback was collated and added to the guidance document to review in round 3.

### Delphi Round 3

Experts were asked to review the revised criteria arising from round 2, together with the findings of the systematic review and anonymized industry feedback. Experts were given an opportunity to suggest removal, merging, splitting, or changes to criteria or their ratings. Through analysis of round 3 responses, essential and recommended consensus criteria were finalized. Criteria for which the response to industry comments was unresolved or conflicting comments were received during round 3 were highlighted for discussion during the consensus meeting.

### Consensus Meeting

Experts were invited to attend the final consensus meeting in person or by web link. The meeting focused on criteria for which consensus had not yet been achieved. Each relevant criterion was discussed until agreement was reached to retain, edit, or remove it from the guidance. The meeting was facilitated by an independent nonvoting chair with experience in BMS regulation, Peter Aggett, MD, PhD (University of Lancaster, UK). Experts were given the opportunity to comment on each criterion, and for those who wished to raise issues anonymously, opportunities were given to submit questions or comments prior to or during the meeting, to be raised by the chair on their behalf. The study team (K.J., B.H., and R.J.B.) circulated minutes after the consensus meeting, and the meeting was recorded. Any final edits and formatting changes were agreed on through email exchange after the meeting.

### Trial Participant and Ethics Committee Consultation

After the consensus meeting, the final criteria were sent to parents of infants who had participated in a BMS trial and to the London Riverside National Health Service Research Ethics Committee for formal comment.

## Results

### Setting and Participants

This Delphi consensus was undertaken between January 1 and September 30, 2018, with a consensus meeting on October 24, 2018. Twenty-eight experts were contacted and 23 participated in at least 1 stage of the Delphi survey: 6 clinical trialists, 9 experts in BMS regulation, 5 clinical trial methodologists, 2 experts in breastfeeding support and infant feeding research, and 1 medical journal editor. Experts were affiliated with institutions in Europe, North America, and Australasia. Sixteen of the experts were able to contribute to the final consensus meeting. Six of 7 invited BMS industry representatives provided comments between June 1 and September 30, 2018, comprising representatives from Danone Nutricia, Nestlé Nutrition, Abbott Nutrition, Hipp, Friesland Campina, and Dairy Goat Co-operative.

### Delphi Survey Results

Initial guidance for round 1 included 73 criteria derived from clinical trials, BMS and breastfeeding guidance, and appraisals of the BMS trial literature. General comments raised in the BMS industry consultation related to overlap with existing clinical trial guidance, the value of study designs other than randomized clinical trials, definitions of BMS and other nutritional products, and the title and scope of the guidance. Preliminary findings from the pilot phase of the systematic review, which evaluated a sample of 61 recent BMS trials, were a lack of independently funded studies and a high prevalence of nonregistered trial outcomes highlighted in publication abstracts.

The outcomes at each stage of the Delphi process are summarized in the Figure. The final guidance comprises 54 essential criteria (eTable 1 in the Supplement) and 4 recommended criteria (eTable 2 in the Supplement). Of these, 18 criteria are specific to BMS trials, which are summarized as a checklist in Table 2.^20,22^ The 58 criteria are elaborated in the eAppendix in the Supplement, including a list of definitions for the key terms used. Key issues discussed at the consensus meeting centered around 4 themes.

**Table 2.** Abbreviated Checklist of Criteria Specific for Clinical Trials of BMS

Domain, item No.^a^	Consensus statement
BMS composition and formulation	
4a	The trial protocol and trial reports clearly describe the composition and formulation of the experimental and control BMS and their relationship, if any, to existing BMS products marketed anywhere in the world
4b	The experimental and control BMS both meet legally required compositional standards, and any instructions for safe reconstitution of BMS by trial participants are consistent with relevant national or international guidance
4c	The trial protocol and trial reports clearly describe any differences between experimental and control BMS which are additional to the constituent(s) of interest and consider their potential impact on the trial results
4d	Appropriate preclinical studies have been performed for previously untested components of BMS
Intervention	
7a	For trials with a primary noninferiority or equivalence objective, such as growth and tolerance trials, participants should be exclusively BMS fed at enrollment
7b	The trial protocol and trial reports describe how intake of experimental and control BMS is recorded during the trial, and the trial reports summarize experimental and control BMS intake in each treatment group during the intervention period
7c	Trial participants’ intake of any foods other than experimental and control BMS during the intervention and data collection periods is recorded
7d	The age of infants at the start and end of the intervention period is appropriate for the trial objectives, and the age range at enrollment is sufficiently narrow for treatment effects to be comparable across the trial population
Outcome assessment	
8c	For growth outcomes, trial reports should comment on whether metabolic and developmental outcomes were also evaluated
Analysis	
12b	Statistical analyses which were not prespecified in the trial protocol are interpreted with caution and are not used as the basis for claims in the trial conclusions, or to support recommendations for infant feeding
Ethics for trials in BMS-fed infants	
14	For trials where participants are all exclusively BMS fed at enrollment, such as growth and tolerance trials, carers’ decision not to breastfeed should be firmly established prior to enrollment in the trial
Ethics for trials where some participants consume breast milk^b^	
15a	The ethics statement in the trial protocol and trial reports clearly states how breastfeeding was supported during the trial
15b	Trial methods do not involve anything that may be interpreted as an incentive to introduce BMS to an infant’s diet and emphasize the superiority of breastfeeding over BMS in all literature
15c	Randomization and treatment allocation do not occur until the time point when a participant expresses an intention to introduce BMS, and participants are offered skilled breastfeeding support from a trained breastfeeding counselor at this stage, prior to randomization and introduction of experimental and control BMS
15d	Incentives to participate in the trial do not include provision of free or discounted BMS, samples, equipment, or other gifts related to BMS and its marketing; if free or discounted BMS is felt to be essential, then a similar level of reimbursement should be provided for continued breast milk feeding^c^
15e	For trials which involve groups of infants at increased risk of a severe adverse event related to BMS use, a high level of scrutiny regarding the possibility of a negative impact on breast milk feeding is required^c^
Limitations	
19c	Trial reports discuss the limitations of any findings which are based on analysis of participants with a minimum level of experimental or control BMS intake^c^
Conflict of interests	
20d	An investigator who is independent of the BMS industry takes overall responsibility for the conduct of the trial, planning and conduct of statistical analyses, decision to publish, reporting, and interpretation of the trial findings, and ensures that the planning and conduct of statistical analyses are led independently of the BMS industry

Abbreviations: BMS, breast milk substitute; GRADE, Grading of Recommendations, Assessment, Development and Evaluations.

^a^ Item No. refers to the full criteria in eTables 1 and 2 in the Supplement. Criteria were scored using the GRADE scale.^20^

^b^ For growth and tolerance trials, or other trials with noninferiority or equivalence objectives, participants should be fully BMS fed and the decision not to breastfeed should be firmly established prior to enrollment in the trial. For other trials, where some participants may be receiving breast milk at enrollment or during the intervention period, trial design and conduct should comply with the International Code of Marketing of Breast-milk Substitutes^22^ and subsequent relevant World Health Assembly resolutions to avoid undermining breast milk feeding.

^c^ Recommended criteria. All other criteria were classified as essential (Table 1).

#### Theme 1: Research Integrity and Reporting Transparency

Experts stressed the importance of transparency of trial conduct and reporting: that all BMS trials are registered; that trial outcomes are made publicly available, in line with current initiatives in medical research that aim to increase access to original data sets^23,24,25^; and that oversight of trial conduct, analysis, and reporting, including adverse event coding, is independent. Independence was conceptualized as usually meaning that trial oversight was the responsibility of the principal investigator, and should not be the responsibility of an employee of the BMS industry or any other entity with a potential financial interest in the outcome of the trial. It was thought that in-house industry-led statistical planning and analysis is not appropriate unless there is complete transparency owing to audit by regulators or full publication of participant-level outcome data, such that all statistical analyses can be independently verified. When blinded BMS products are used as trial interventions, industry collaboration may be necessary, but trialists and BMS manufacturers should avoid creating financial dependencies and avoid industry control of trial conduct, analysis, or reporting. The TRIGR (Trial to Reduce Insulin-Dependent Diabetes Mellitus in the Genetically at Risk) study was cited as a good example of “arm’s length” BMS trial practice, in which the BMS manufacturer’s role was limited to provision of trial interventions.^26^ Experts also emphasized that significant trial amendments—especially changes to participant inclusion criteria, experimental or control treatment, and methods, timing, or nature of outcome measures—should be recorded by way of an update to the BMS trial’s record on a World Health Organization–approved clinical trial registry.

#### Theme 2: Study Design and Breastfeeding Support

The provision of breastfeeding support in BMS trials was a controversial area, resolved by experts through identifying the importance of distinguishing 2 different approaches to breastfeeding support for 2 different types of studies. In BMS trials designed to meet a noninferiority or equivalence objective—typically those aiming to demonstrate adequate infant growth and tolerance of a new BMS product—experts thought that participating infants should be fully BMS fed and the decision not to use breast milk should be firmly established prior to enrollment in the trial. After randomization, additional breastfeeding support is not usually required for participants in these studies, but it is important to ensure that appropriate breastfeeding support has been provided prior to enrollment. In some countries, regulators have additional specific requirements for infant growth and tolerance trials—for example, in the United States, growth trials must enroll infants at age 14 days or younger with an intervention period that lasts for 15 weeks or more.^27^ These noninferiority or equivalence trials should usually be analyzed using both intention-to-treat and a prespecified per-protocol data set.

In a separate group of BMS trials, usually pragmatic superiority trials aiming to generate data to support a nutrition or health claim, some infants are receiving breast milk at enrollment. Superiority trials should usually be analyzed using an intention-to-treat data set. In trials in which some infants are receiving breast milk at enrollment, experts agreed that it is important to demonstrate adequate support for breast milk feeding within the trial. In these studies, it was thought that an international board–certified lactation consultant employed by an academic or health care institution would be best placed to offer skilled breast milk feeding support.

#### Theme 3: Description of Trial Interventions

Experts confirmed the scope of this guidance as being BMS, as defined by the World Health Organization, including all ingredient additives to BMS that are delivered to an infant within a BMS. Experts agreed that composition and formulation of the experimental and control BMS need to be fully described and related to existing marketed products, and that the timing of the intervention period should be appropriate for the trial objectives. Trial participants’ intake of both experimental and control BMS and any other foods should be accurately recorded.

#### Theme 4: Study Outcomes

Experts agreed that primary and secondary study outcomes should be clearly established a priori and that statistical power calculations for the primary outcome should be based on a clinically meaningful effect size. The end points used to measure each outcome should be valid and clinically relevant, and the use of surrogate end points in place of clinical end points should be appropriately justified and interpreted.

### Trial Participant Viewpoints

After the consensus meeting, 16 BMS trial participants were contacted and 5 responded, with 3 providing detailed commentary and telephone discussion regarding the criteria. All responding BMS trial participants were supportive of the final criteria, especially independence of trial conduct and analysis and transparent reporting of outcomes. The BMS trial participants commented in detail on 2 criteria concerning the subset of BMS trials in which some infants are receiving breast milk at enrollment. These criteria (15c and 15d) are not relevant to trials in which infants are exclusively fed BMS prior to enrollment and the parents’ decision to not provide breast milk is firmly established prior to enrollment. In support of criterion 15c, they thought that provision of trial BMS should not occur until randomization, and that this provision should not occur during pregnancy or (where relevant) during exclusive breast milk feeding, to avoid providing an incentive to use BMS in place of breast milk. However, participants thought that once a parent decides to supplement breast milk feeding with a BMS, the use of other BMS products should be permitted prior to provision of trial BMS, to avoid feeding problems while awaiting delivery of the experimental or control BMS. In relation to criterion 15d, BMS trial participants viewed the provision of free trial BMS as useful, and supportive for participants with financial constraints, but recognized that this provision may incentivize breastfeeding women to use BMS in place of breast milk. One participant suggested that if free BMS is provided in a trial that includes breastfed infants, a financial incentive to continue breastfeeding could also be provided. The experts agreed by email to add this suggestion to criterion 15d.

## Discussion

Clinical trials of BMS require specific guidance to ensure that they are methodologically sound, such that their results may reliably inform caregivers and health care professionals. This Delphi survey has derived, through expert consensus, a standard consisting of 58 criteria to support the design, conduct, analysis, transparent reporting, and evaluation of BMS trials. Implementation of this standard, in conjunction with existing methodological and ethical guidance, could better protect BMS trial participants and ultimately improve the quality of BMS products and information associated with them for consumers.

The validity of this Delphi process is supported by the extensive review of relevant sources that informed the initial criteria and the engagement of a comprehensive panel of experts who provided a diverse range of experience and insight. The consistent and anonymous application of each iteration, as defined a priori in the protocol, minimized bias and manipulation of experts’ opinions. Outcomes from analysis of a sample of BMS trials identified by a pilot systematic review usefully informed the Delphi process. The inclusion of a face-to-face consensus meeting resolved any remaining issues. It was not possible to maintain anonymity of experts at this stage, but the meeting was carefully moderated by an independent chair, through whom experts were invited to submit questions or issues anonymously. Although only 13 of 23 Delphi experts attended the meeting, 3 others provided written comments that were considered during the meeting; a full summary of the discussions and decisions, and then the final manuscript, were shared with all experts for comment and approval after the meeting. One expert withdrew from authorship of the article because of disagreement with specific criteria, although these met the predefined requirements for consensus summarized in Table 1.^20^ To limit bias introduced during development of the criteria, the study team reproduced all experts’ comments anonymously and verbatim in each round. Industry representatives were asked to comment, but not to score the criteria.

### Limitations

This study had some limitations. We had good representation from Europe and North America, where most BMS trials are conducted, but less good representation from other regions where BMS trials are less commonly conducted. We did not involve industry in the whole Delphi process, because that would represent a conflict of interest for some experts in relation to their regulatory work. This new guidance therefore represents the views of trialists, methodologists, lactation consultants, infant feeding researchers, regulators, and a journal editor rather than the views of industry representatives. Parents of infants who had participated in a BMS trial commented on the criteria at the final stage but were not members of the Delphi panel and did not score criteria.

## Conclusions

We have developed new, consensus-based guidance for the design, conduct, analysis, and reporting of BMS trials. To achieve our aim of improving the conduct and reporting of BMS trials, this guidance must come to represent the expected standard in this field. Industry representatives, regulators, and clinical trialists have been able to contribute their views on the feasibility and practicality of these criteria, and some regulators such as Health Canada have already incorporated the criteria into their guidance.^28^ If BMS trialists incorporate this guidance in their clinical trials, in conjunction with existing methodological and ethical guidance, the quality and validity of their trials will benefit, so participants will be protected and the infant nutrition communitywill be better informed about the safety and potential efficacy of BMS products.

## Notes

Supplement. **eAppendix.** Definitions, eMethods, and eResults **eTable 1.** Essential Criteria for the Conduct and Reporting of Clinical Trials of Breastmilk Substitutes **eTable 2.** Recommended Criteria for the Conduct and Reporting of Clinical Trials of Breastmilk Substitutes **eReferences.**
