# Supplementary material for: Guidance for the Conduct and Reporting of Clinical Trials of Breast Milk Substitutes
Source: JAMA Pediatr. 2020 May 11;174(9):874–81. doi: 10.1001/jamapediatrics.2020.0578 (PMC7215627; doi:10.1001/jamapediatrics.2020.0578)
Supplement: Supplement. — eAppendix. Definitions, eMethods, and eResults eTable 1. Essential Criteria for the Conduct and Reporting of Clinical Trials of Breastmilk Substitutes eTable 2. Recommended Criteria for the Conduct and Reporting of Clinical Trials of Breastmilk Substitutes eReferences. [file jamapediatr-e200578-s001.pdf]

Supplementary Online Content

Jarrold K, Helfer B, Eskander M, et al. Guidance for the conduct and reporting of clinical trials of breast milk substitutes. *JAMA Pediatr.* Published online May 11, 2020. doi:10.1001/jamapediatrics.2020.0578

eAppendix. Definitions, eMethods, and eResults
eTable 1. Essential Criteria for the Conduct and Reporting of Clinical Trials of Breastmilk Substitutes
eTable 2. Recommended Criteria for the Conduct and Reporting of Clinical Trials of Breastmilk Substitutes
eReferences.

This supplementary material has been provided by the authors to give readers additional information about their work.

© 2020 Jarrold K et al. *JAMA Pediatrics*.

eAppendix. Definitions, eMethods, and eResults

Key Definitions

Breastmilk substitute (BMS): in accordance with the World Health Organization's definition, we define BMS as any food intended as a partial or total replacement for breastmilk, and marketed for children. This definition includes exempt infant formulas or formulas for special medical purposes as well as supplements or fortifiers added to BMS, but excludes nutritional interventions such as vitamin or mineral supplements which are not given within a BMS. Any clinical trial which includes a substance which is added to a BMS, and therefore becomes part of BMS prior to ingestion by the infant, is considered a BMS trial and is covered by this guidance. Products given separately to BMS are not covered by this guidance. Human milk products such as stored mother's milk, pasteurised donor milk or human milk fortifier may sometimes be used as total or partial substitutes for breastfeeding. Although we have not explicitly addressed human milk products in this guidance, those undertaking clinical trials of human milk products should carefully consider which aspects of this guidance are relevant to their trial. The guidance is intended to cover trials which enrol at least one participant prior to their first birthday.

Clinical endpoint: a characteristic or variable that reflects how a patient feels, functions (including growth and behaviour), or survives [1].

Clinical trial: a research study that prospectively assigns human participants or groups of humans to one or more health-related interventions, to evaluate the effects on health outcomes. Clinical trials usually, but not always, involve random assignment to interventions. This new guidance was developed with the most common BMS trial design in mind: a randomized controlled parallel-group clinical trial, with an equivalence or non-inferiority design for growth and tolerance trials and a superiority design for efficacy trials. Observational studies and post-marketing surveillance studies are not covered by this guideline. Other clinical trial designs such as cross-over, cluster randomized or stepped-wedge trials have not been fully addressed, but some aspects of the guidance will be relevant such as the ethics, breastfeeding support, conflict of interest and trial reporting sections. Future BMS guidance should provide additional criteria tailored to observational studies and alternative trial designs.

Clinical trial register: a register which conforms to World Health Organization standards [2]. Eligible registries are both the Primary Registries and the Partner Registries listed in the World Health Organization Registry Network https://www.who.int/ictrp/network/en/

Composition: specification of nutritional components of a given food including a list of ingredients and the food's nutrient profile (amount of each essential nutrient in the product).

Efficacy trial: a clinical trial evaluating the effect of feeding an experimental BMS when compared to a control BMS, usually a currently marketed and approved BMS.

Equivalence analysis: statistical analysis of a specific trial outcome which has the objective of showing that the effect of the experimental BMS on the outcome is similar to the effect of a control BMS intervention (usually a currently marketed and approved BMS) on the same outcome. Usually an 'equivalence margin' is set by trial investigators.

Formulation: the preparation and combination of ingredients, including the form of ingredients (e.g. type of protein), and the matrix (e.g. solution) in which ingredients are delivered. Formulation can alter nutritional bioavailability and biochemical or immunological properties of the product [3].

Growth and tolerance trial: a clinical trial evaluating safety and nutritional adequacy of feeding an experimental BMS to support normal physical growth, when compared to a control BMS, usually a currently marketed and approved BMS.

Independent: in the context of taking overall responsibility for a BMS trial, or undertaking specific responsibilities such as data analysis, adverse event categorisation or breastfeeding support, 'independent' means that individuals should have primary affiliations to a hospital or clinic, or hospital- or clinic-affiliated clinical trials or biostatistics unit, or a university. Employees of BMS industry or any other entity with a potential financial interest in the outcome of a trial are not 'independent'. Data Safety and Monitoring Boards have specific requirements for independence, set by the International Council for Harmonisation of Technical Requirements for Pharmaceuticals for Human Use [4]

© 2020 Jarrold K et al. *JAMA Pediatrics*.

Intention to treat data: data for all participants who were randomly allocated to a treatment are included in the analysis and participant data are analysed in the same groups to which the participants were randomised, whether or not they actually received or completed the allocated treatment regimen.

Non-inferiority trial: a trial with the main objective of showing that the effect of the experimental BMS on the primary outcome is not inferior to the effect of a control BMS intervention on the same outcome. Usually a 'non-inferiority margin' is set by trial investigators, for example they might aim to show that a trial intervention does not reduce weight gain by more than a specific number of grams per day during a period of trial BMS feeding [5,6].

Outcome measure: an efficacy or safety or other measure in a clinical trial, evaluated using a specific endpoint [7].

Per protocol data: the set of data generated by the subset of participants who complied with the trial protocol sufficiently to ensure that these data would be likely to exhibit the effects of the experimental BMS, according to the underlying scientific model. Inclusion in the per protocol dataset requires consideration of exposure to treatment, availability of measurements and absence of major protocol violations.

Significant amendment: a change to the Trial Protocol which significantly affects one of the following: the safety or physical or mental integrity of study participants; the conduct or management of the study; the scientific value of the study; the quality or safety of any investigational medicinal product used in the study.

Superiority trial: a trial with the main objective of showing that the effect of the experimental BMS on the primary outcome is superior to the effect of a control BMS intervention on the same outcome.

Surrogate endpoint: a variable that provides an indirect measurement of effect in situations where direct measurement of clinical effect is not feasible or practical. For example a laboratory or physical measurement that does not directly represent clinical effect [8], but is assumed to predict a clinical endpoint based on scientific evidence [1].

Trial protocol: a document published prior to the trial report describing trial objectives, design, statistics, methodology and organization, as well as trial background and rationale [4].

Trial report: a report describing trial methods and outcomes, submitted to a regulator or to a peer-reviewed journal.

© 2020 Jarrold K et al. *JAMA Pediatrics*.

eMethods

Scope of this Guidance

This guidance is designed to complement, rather than replace, other clinical trial guidance such as The International Council for Harmonisation Good Clinical Practice Guidelines [4], other trial checklists such as CONSORT (Consolidated Standards of Reporting Trials) Statement [9] or other guidance specific to BMS trials such as that published by the US Food and Drug Administration or European Food Standards Agency [10-16]. The guidance aims to help those designing, conducting, analysing or reporting clinical trials of BMS, sponsors of BMS trials and those who need to evaluate or critically appraise BMS trial protocols and reports. While regulators often produce their own guidance for BMS trials which may be used as part of a regulatory dossier, this guidance is likely to be useful both for those evaluating regulatory dossiers and those evaluating journal manuscripts. The guidance is relevant for all clinical intervention trials of a BMS undertaken in human infants enrolled prior to their first birthday, but some criteria within the guidance are only relevant to specific types of BMS trials. An important distinction is between growth and tolerance trials of a new BMS product with non-inferiority or equivalence objectives, where it is usual and recommended to only enrol BMS fed infants where the decision to not breastfeed is firmly established; and other BMS trials with superiority objectives, for example those aimed at providing evidence in relation to a health or nutrition claim, where infants who are receiving breastmilk at the time of enrolment may be included. Several of the final consensus criteria, relating to breastfeeding support, are relevant for the latter type of trial, but not relevant for most growth and tolerance trials.

This guidance provides a set of criteria to help design or evaluate a BMS trial, which relevant stakeholders have agreed are important. The criteria do not cover all details of a BMS trial, and there is a need for more detailed guidance for the development of BMS Trial Protocols. For example, growth and tolerance trials, which are often a regulatory requirement prior to marketing of a new BMS, require separate specific guidance in order to harmonise practice across trials. The optimum growth endpoints need to be defined, and the optimal methods of endpoint measurement and acceptable limits of variation for different populations are just some of the areas which need detailed guidance. Some specific guidance for growth trials has been published by national regulators, for example the United States Food and Drug Administration's "Requirements for quality factors for infant formulas" and "Quality factor assurances for infant formulas" as well as Health Canada's 'Protocol for a Growth/Tolerance Clinical Trial for Healthy Term Infants' [5,6].

Definition of Delphi Consensus

The consensus outcome definition we used (which is summarised in Table 1 of the manuscript) is adapted from the MOMENT study protocol [17]. The threshold required to reach consensus is subjective and varies among Delphi studies, with greater weight attributed to the importance of defining these cut offs *a priori* to minimise bias. If an expert did not give a score for a criterion ('unable to score'), the percentage required to meet the consensus definition was unchanged. 'Essential' and 'recommended' criteria must therefore have been scored highly by the majority of experts, with only a small number scoring them as 'not important at all' or 'unable to score'; whereas the reverse must be true of criteria that the consensus determines should be excluded.

© 2020 Jarrold K et al. *JAMA Pediatrics*.

Delphi Panel Selection

The Delphi method does not mandate a minimum sample size but requires a panel of experts that are representative of all relevant professional groups and stakeholders. Therefore, experts in BMS trials designed to demonstrate adequate growth and tolerance, in BMS trials with other objectives such as supporting health and nutrition claims, in BMS regulation, trial methodology, breastfeeding support, infant feeding research and medical publishing were identified through consultation with others working in these fields. Each expert invited was asked to nominate other relevant experts to contribute. The objectives and methods were explained through circulation of the study protocol. Consent was implied through ongoing involvement in the Delphi process. Each round of the Delphi survey was piloted by the study team prior to initiation, and experts were given 3-4 weeks to complete each round, with regular prompts to maximise participation. Only experts completing each round of the process were eligible to take part in subsequent rounds, except where new experts were nominated by an existing expert as a substitute in a later round, or where contractual obligations prevented expert from commenting on initial rounds.

Development of Initial Guidance for Delphi Round 1

Initial criteria were developed through a review of existing guidance for the conduct of clinical trials, specific guidance for trials of BMS, BMS legislation, BMS marketing guidance, BMS regulatory requirements and published critical appraisals of BMS trials. The following sources were reviewed and their recommendations extracted during the development of the initial guidance document, which was circulated to experts in round 1: CONSORT (Consolidated Standards of Reporting Trials) Statement [9], The Declaration of Helsinki [18], The International Council for Harmonisation Good Clinical Practice Guidelines [4], International Committee of Medical Journal Editors (ICMJE) Recommendations [19], The Cochrane Collaboration Risk of Bias Tool [20], The WHO International Code of Marketing of Breast-Milk Substitutes [21], European and North American BMS legislation [10,12], Codex compositional requirements for BMS [22], and European Food Safety Authority (EFSA) and United States Food and Drug Administration (FDA) regulatory requirements [13-16].

Systematic review

A pilot systematic review of a sample of recent BMS trials was undertaken to evaluate the adherence of published studies to the proposed criteria and identify any new methodological issues in BMS trials. The systematic review was registered on PROSPERO (CRD42018091928) and the final report of the full systematic review will be published separately [23]. Preliminary findings from the pilot phase of the systematic review were summarised for experts prior to completion of Round 3.

© 2020 Jarrold K et al. *JAMA Pediatrics*.

eResults

This guidance applies to all clinical intervention trials of BMS undertaken in human infants enrolled prior to their first birthday. These trials may have a non-inferiority or equivalence primary outcome, for example growth and tolerance trials; or may have a superiority primary outcome, for example trials designed to generate evidence in support of a health or nutrition claim. Some criteria apply equally to these types of trials, but others are specific to one type of trial, and this is indicated below where relevant.

1. Trial Design and Rationale

1a. The Trial Protocol and Trial Reports include a summary of the existing evidence in the area of study, and an explanation of the trial rationale.

The summary might include evidence from previous randomised control trials, observational studies, animal or *in vitro* studies. For growth and tolerance trials evaluating a new or a significantly changed BMS product, the explanation should include a clear description of the composition and formulation of the BMS in relation to established national or international standards. For efficacy trials which might potentially support a health claim, the explanation should also include description of a plausible biological relationship between a BMS component or modification and a proposed health benefit.

1b. The Trial Protocol and Trial Reports include a clear description of the trial design, objectives and hypotheses.

This description should especially make clear whether the primary trial objective is a growth and tolerance trial, efficacy trial, whether the trial has combined objectives, or has objectives which do not relate to growth, tolerance or efficacy.

1c. The trial design is appropriate for the trial objectives, specifying whether the trial is a superiority, non-inferiority or equivalence trial.

This means that the trial design will usually be a randomised controlled parallel-group design. For most efficacy trials this should be a superiority design. In some trials, especially growth and tolerance trials, an equivalence or non-inferiority design may be appropriate, with a clearly stated predefined equivalence or non-inferiority margin. Superiority, equivalence and non-inferiority design is defined as above, based on study objectives and the intended type of statistical analysis used for the trial primary outcome.

2. Trial Registration

2a. The Trial Protocol is registered on a clinical trial register which includes all information specified in the World Health Organization Trial Registration Data Set.

The Trial Protocol should be registered prior to enrolment of the first trial participant, on a clinical trial register which conforms to World Health Organization standards [2]. The WHO International Clinical Trials Registry Platform (ICTRP) is available at WHO website [24]. The version of the Trial Registration Data Set that is current on the date that the Trial Protocol was registered should be used. Examples of suitable trial registries are available on the WHO website [25]. The registration number and name of the trial registry should be clearly described in the Trial Protocol and Trial Reports.

2b. Trial Reports clearly describe any amendments to the protocol after trial commencement, and the clinical trial register is updated when significant amendments are made.

When a significant amendment to the Trial Protocol occurs, especially a change to participant inclusion criteria, experimental or control treatment, or methods, timing or nature of endpoint assessment, this should be recorded by way of an update to the BMS trial's record on a World Health Organization approved clinical trial registry. It should be noted that any deviation from Trial Protocol, such as any change in trial conduct or pre-planned analyses, should also be described in the Trial Report, together with timing, reasons for the change, persons responsible and implications of the change. This should be done regardless of whether the deviation was a formal change to the Trial Protocol or not. Personnel changes are usually not documented as a formal change to the Trial Protocol [8].

2c. The Trial Protocol clearly defines any trial stopping rules and withdrawal criteria.

An independent Data Safety Monitoring Board (DSMB) should be established prior to enrolment of the first participant. Part of the DSMB's role is to recommend stopping the trial when necessary. Trial stopping rules should be defined separately for individual trial participants and for the whole trial or parts of the trial [4]. The DSMB should have written operating procedures and maintain records of all its meetings, including interim result analyses. It should include clinical trial scientists knowledgeable in the field and statistical expertise.

3. Recruitment

3a. The Trial Protocol and Trial Reports clearly describe the pre-defined inclusion and exclusion criteria which guided participant recruitment.

© 2020 Jarrold K et al. *JAMA Pediatrics*.

Details of each inclusion and exclusion criterion should be recorded, and the settings and locations where the participants were recruited should be included in the Trial Reports.

3b. The Trial Protocol and Trial Reports describe the method of participant recruitment in detail.

Description of the recruitment methods needs to include the methods used to ensure that trial participants were not coerced into taking part in the trial at any of the trial sites.

4. BMS Composition and Formulation

4a. The Trial Protocol and Trial Reports clearly describe the composition and formulation of the experimental and control BMS and their relationship, if any, to existing BMS products marketed anywhere in the world.

Authors should specify the ingredients, the quantity of each ingredient, the nutrient profile, the formulation, and where appropriate the ratio of ingredients to each other, for the experimental and control BMS. Authors should specify the composition of any ingredient(s) that differ between the experimental and control BMS, including the quantity, concentration, form and source of the ingredient(s). Some of the information about composition or formulation may need to be included as an appendix or supplemental file to the document, depending on specific formatting requirements. If a BMS is currently available anywhere in the world, or is a modification of a currently available product, this should be clearly documented.

4b. The experimental and control BMS both meet legally required compositional standards, and any instructions for safe reconstitution of BMS by trial participants are consistent with relevant national or international guidance.

While both experimental and control BMS should usually meet legally required compositional standards such as those derived from Codex standards [22], there may be trials where the experimental BMS includes a change to legally required compositional standards. This may be acceptable if appropriately justified and if the control BMS meets legally required compositional standards. The decision as to whether the experimental and control BMS meet the legally required national and international standards may be made by a research ethics committee and/or a regulatory authority. If the experimental BMS is not already marketed, then careful consideration of potential safety issues is required, which may include review of data from animal studies. For all BMS trials, the control BMS should usually be approved and marketed for use in the local population prior to enrolment of the first trial participant.

4c. The Trial Protocol and Trial Reports clearly describe any differences between experimental and control BMS which are additional to the constituent(s) of interest and consider their potential impact on the trial results.

For growth and tolerance studies, since the objective is usually to show that the experimental BMS is either equivalent to, or not inferior to, the control BMS (non-inferiority trial), the control BMS should be a commercial BMS that has already been clinically tested and proven safe and nutritionally adequate. For efficacy trials with a superiority objective which aim to provide evidence which might support a nutrition or health claim, the composition and formulation of the experimental and control BMS should be similar except for the component(s) of interest which are relevant to the claim.

4d. Appropriate pre-clinical studies have been performed for previously untested components of BMS.

This is particularly important where these previously untested components will be used in a trial involving children aged less than 15 weeks. These components must be tested for safety using an appropriate approach which is consistent with regulatory guidance, for example the EFSA Scientific Committee Guidance [26] or Generally Recognized As Safe (GRAS) by the Food and Drug Administration [27].

5. Randomisation

5a. Participants are randomly allocated to either the experimental or control group, and the procedure for randomisation is clearly described.

Acceptable procedures include computer-generated randomisation lists, which may include stratification and block randomisation. Unacceptable procedures include alternation or randomisation according to clinic, date of birth or attendance, or other participant features.

5b. Personnel involved in enrolling participants and assigning interventions are blinded to the allocation sequence, and methods used to conceal allocation are clearly described.

Acceptable procedures include third-party allocation by a clinical trial pharmacist or remote trial co-ordinator or use of sequential, opaque, sealed envelopes or randomisation software. Unacceptable procedures include allocation using methods where the randomisation sequence can be anticipated, for example allocation by a trial clinician who has open access to the full randomisation list.

© 2020 Jarrold K et al. *JAMA Pediatrics*.

6. Blinding

6a. The Trial Protocol and Trial Reports clearly describe methods to achieve blinding of participant carers, or an explanation of why blinding is not feasible.
The experimental and control BMS products should look, smell and taste identical, where feasible, and should be packaged identically, with the exception of a coded package label which allows linkage to the randomisation list. In some circumstances, despite the use of identical packaging, the experimental and control BMS products may differ in appearance, smell and/or taste. In such circumstances, where blinding of participants' carers may not be feasible, the Trial Protocol and Trial Reports should include at least one objective outcome measure, the reporting of which cannot be directly influenced by participants' carers, e.g. growth parameters, faecal or urinary markers, or where measurement is objective and can be undertaken by individuals who remain blind to treatment allocation.
6b. The Trial Protocol and Trial Reports clearly describe the method by which outcome assessors are kept blind to which treatment group participants were allocated throughout the trial.
For each outcome measure there should be sufficient information presented to demonstrate whether outcome assessment was undertaken with or without knowledge of treatment allocation.

7. Intervention

7a. For trials with a primary non-inferiority or equivalence objective, such as growth and tolerance trials, participants should be exclusively BMS fed at enrolment.
These trials should include a planned analysis of those infants who received at least 15 weeks of exclusive BMS intake, starting not later than 14 days of age [5]. If this duration is not possible, the Trial Protocol and Trial Reports should justify why evaluation of a shorter intervention period may be appropriate.
7b. The Trial Protocol and Trial Reports describe how intake of experimental and control BMS is recorded during the trial, and the Trial Reports summarise experimental and control BMS intake in each treatment group during the intervention period.
If the trial includes a planned analysis of a subset of infants with a specific intake of experimental BMS within a specific time period, for example a Per Protocol analysis, then criteria for being included in this subset are clearly described in the Trial Protocol and Trial Reports.
7c. Trial participants' intake of any foods other than experimental or control BMS during the intervention and data collection periods is recorded.
It is important to record whether or not any foods other than experimental or control BMS were used by trial participants during the intervention and data collection periods of the trial. The record of other dietary intake includes, where relevant, BMS intake other than experimental and control BMS, breastfeeding duration, timing of complementary food introduction, and the quantity and type of complementary food and nutrient intake, including use of nutritional supplements.
7d. The age of infants at the start and end the intervention period is appropriate for the trial objectives, and the age range at enrolment is sufficiently narrow for treatment effects to be comparable across the trial population.
The age of infants at the start and end of the intervention period is appropriate with regard to the objective(s) of the trial, and ensures that the age range of participants at enrolment is sufficiently narrow for treatment effects to be comparable across the trial population. For example, as stated under 7a, growth and tolerance trials designed to demonstrate non-inferiority or equivalence should aim to enrol participants age 14 days or younger.

8. Outcome assessment

8a. The Trial Reports clearly state the pre-defined specific primary and secondary outcomes, including timing and method of outcome assessment, and these are consistent with the clinical trial register record.
If there is a change to outcome assessments after trial registration, this should be recorded in the trial registry as per criterion 2b.
8b. The endpoints assessed for each outcome include recognized, well-defined, measurable, interpretable, valid, reliable and clinically relevant endpoints.
The endpoints assessed for each outcome include a recognized, widely-accepted measure of the outcome in question, where such measures exist, and are evaluated at appropriate time-points. When applicable, any methods used to enhance the quality of the measurements (e.g., training of assessors) should be reported. For example, growth outcomes might use World Health Organization growth standards [28] or where widely-accepted Core Outcome Sets exist, such Core Outcomes might be used. This criterion applies to all registered outcomes, both primary and secondary. While some trials might include outcomes which are not directly clinical, they should at least be potentially relevant to a clinical outcome.
8c. For growth outcomes, Trial Reports should comment on whether metabolic and developmental outcomes were also evaluated.

© 2020 Jarrold K et al. *JAMA Pediatrics*.

This is because growth outcomes alone may not be adequate measures of nutritional suitability, since developmental or metabolic status can be affected by nutritional exposures without detectable effects on weight gain [29].

9. Surrogate endpoints

9a. Any surrogate endpoints used to predict the outcome are appropriate, clinically relevant, measurable, interpretable and widely-accepted measurements.
Appropriate surrogate endpoints might include, for example, biochemical vaccine-specific immunoglobulin G levels as a surrogate for clinical protection from vaccine-relevant disease following vaccination. Inappropriate surrogate endpoints might include correlates of biological activity which have not been shown to be mechanistically and causally associated with the clinical endpoint. For example, measures of faecal microbiome have not been established as mechanistically and causally associated with intestinal symptoms such as infant colic or gastroesophageal reflux [30,31].
9b. If a surrogate endpoint is used, the Trial Protocol and Trial Reports discuss the rationale for using a surrogate endpoint in place of a clinical endpoint, and any uncertainty incurred.
Surrogate endpoints might potentially include an exploratory, novel biomarker, if the biomarker is an outcome in the Trial Protocol. Such biomarkers should have a plausible biological relationship with the trial intervention, and their measurement should be accompanied by the measurement of clinical endpoints in the same trial. Trial Reports should include a discussion of any uncertainty around the clinical relevance of findings related to surrogate endpoints or novel biomarkers. Data from any surrogate outcomes should be carefully interpreted, especially if results from surrogate outcomes conflict with any clinical outcomes in the same study.

10. Adverse events

10a. The Trial Protocol includes a valid and well recognized definition of common and anticipated adverse events.
Where appropriate, standardized criteria or validated scales for diagnosis should also be included.
10b. The method for evaluating, categorising and reporting adverse events is independent of individuals or institutions with a potential commercial interest in the outcome of the trial.
The method for evaluating, categorising and reporting adverse events should be specified in the Trial Protocol. This should be undertaken by an individual with a primary affiliation to a hospital or clinic, or hospital- or clinic-affiliated clinical trials or biostatistics unit, or a university. This will usually be conducted by the lead investigator of the trial, who like all investigators is required to comply with the principles of Good Clinical Practice and other relevant regulatory requirements. The lead trial investigator may delegate some of the key decision making to other independent entities, e.g. a DSMB for evaluating and categorising adverse events.
10c. The method for collecting information about adverse events includes a combination of checklists and open-ended questions.
Adverse event recording should also include validated scales or scores, where relevant and available.

11. Statistical considerations

General statistical considerations for clinical trials are described in the International Council for Harmonisation (ICH) guidance ‘Statistical Principles for Clinical Trials’ [4]. The criteria below are consistent with ICH principles.
11a. The Trial Protocol and Trial Reports include a description of how the sample size was determined and the statistical power of the trial, including expected attrition rates.
This description should include details of the timing of any interim analyses, numbers of participants to be enrolled at each trial centre, planned sample size, sample power and clinical relevance, statistical significance level and approach to handling missing data [4].
11b. Statistical power is based on detection of an effect size for the primary outcome(s) which is both statistically significant and clinically meaningful.
While some regulatory bodies may not always require demonstration of clinical significance, and some trial outcomes may not be direct measures of clinical response, trials should generally aim to detect an effect which is clinically meaningful. For surrogate outcomes trials should aim to detect an effect which has the potential to be clinically meaningful.
11c. Statistical methods used to compare groups for the primary and secondary outcomes are appropriate and pre-specified in the Trial Protocol.
For superiority trials, the statistical analyses should use Intention To Treat (ITT) data. For equivalence or non-inferiority trials, statistical analyses should use both Per Protocol (PP) and ITT data. The PP population should be pre-specified in detail in the Trial Protocol. For example, for a 15-week feeding trial, criteria that should be met by BMS fed infants to qualify for the PP analysis might include: completion of the 15-week trial with no more

© 2020 Jarrold K et al. *JAMA Pediatrics*.

than a specific number of days where all feedings were non-study BMS; and consumption of no more than a specific number of non-study BMS feedings during the 15-week trial. The classification into ITT or PP populations should be conducted prior to the database lock, and all clinical primary or secondary non-inferiority or equivalence endpoints should be subject to both ITT and PP analyses to assess the robustness of the results.

11d. The Trial Protocol and Trial Reports describe all pre-specified subgroup analyses and/or adjusted analyses.

The methods for adjusted analyses should be clearly described, including handling of missing data and multiple comparisons.

12. Post-hoc analyses

12a. In the Trial Reports, any additional exploratory statistical analyses are described, acknowledged as *post hoc* analyses and justified.

Any outcomes or analyses which were not pre-specified in a registered Trial Protocol or in a Statistical Analysis Plan which was finalised prior to database lock and the initiation of statistical analysis should be considered *post hoc*.

12b. Statistical analyses which were not pre-specified in the Trial Protocol are interpreted with caution and are not used as the basis for claims in the trial conclusions, or to support recommendations for infant feeding.

The only non-prespecified outcomes which would be appropriate to include in trial or abstract conclusions are serious unexpected adverse events potentially attributable to a trial intervention, which raise a safety concern about a trial intervention. Serious adverse events are life threatening, result in death, require hospitalization or result in disability or incapacity. They are unexpected if the nature or severity of the event is inconsistent with available product information [4]. By 'claims' here we mean claims of a favourable outcome made in the Trial Report, whether or not such claims are used to support a nutrition or health claim for a marketed product. We acknowledge that the status of claims of a favourable outcome and their relevance to applications for regulatory approval will vary across different legislatures.

13. Trial Ethics for all trials

13a. The Trial Protocol and Trial Reports include a statement regarding the trial's adherence to the Declaration of Helsinki and Good Clinical Practice guidance.

A statement to this effect is already required by many leading medical journals.

13b. Trial Reports include an International Committee of Medical Journal Editors (ICMJE) ethics statement.

The ICMJE statement should clearly state that the trial was approved by an institutional review board or research ethics committee independent of the trial funder, and that written informed consent was obtained from caregivers of all participants prior to their infant's enrolment in the trial.

14. Trial Ethics for trials where no participants are receiving breastmilk at enrolment or during the intervention period

Compliance with the International Code of Marketing of Breastmilk Substitutes and Subsequent Relevant World Health Assembly Resolutions

In most trials with a primary non-inferiority or equivalence aim, such as growth and tolerance trials of a new or a significantly changed BMS product, investigators aim to enrol infants who are completely BMS fed, and this is supported by criterion 7a. In these trials it is important to demonstrate that the decision not to breastfeed was firmly established prior to enrolment in the trial, since enrolment of infants receiving breastmilk into a trial of exclusive BMS feeding would violate the Code.

14. For trials where participants are all exclusively BMS fed at enrolment, such as growth and tolerance trials, carers' decision not to breastfeed should be firmly established prior to enrolment in the trial.

In order to demonstrate that the decision not to use breastmilk was firmly established prior to enrolment, investigators should consider excluding infants who have been fed breastmilk within 3 days prior to enrolment. Participant use of a BMS other than experimental or control BMS prior to enrolment should therefore be permitted within the Trial Protocol.

© 2020 Jarrold K et al. *JAMA Pediatrics*.

15. Trial Ethics for trials where some participants are receiving breastmilk at enrolment or during the intervention period

Compliance with the International Code of Marketing of Breastmilk Substitutes and Subsequent Relevant World Health Assembly Resolutions

In some trials with a primary superiority aim, especially pragmatic trials, some participating infants may be partly or completely fed breastmilk, from either their own mother or a breastmilk donor, at the time of enrolment and/or during the intervention period. Participants in such studies require special attention regarding breastfeeding support, due to the concern that recruitment or study procedures might violate the Code or lead to reduced breastmilk exposure. For example, participant information in trials might focus on BMS and potential health benefits of experimental BMS without emphasising the health benefits of breastfeeding, and thereby fail to fully support breastfeeding in potential participants who wish to continue breastfeeding; or trials might provide incentives to introduce BMS in place of breastmilk through provision of free BMS or provision of BMS during pregnancy or exclusive breastfeeding. The following section is relevant to trials which include infants receiving breastmilk at enrolment or during the intervention period, but is not relevant to trials which only include BMS-fed infants where the decision to not breastfeed is firmly established prior to enrolment. Inclusion of infants receiving breastmilk would not usually be appropriate in a non-inferiority or equivalence trial such as a growth and tolerance trial of fully BMS fed infants. Regarding the inclusion of a 'breastfed reference group' the panel did not reach consensus; therefore, in the final guidance there is no criterion that inclusion of such group is essential or recommended for BMS trials. This is because some panel members stated that causal inferences cannot be reliably drawn from comparisons between a non-randomised group of breastfed infants and populations of infants randomised to BMS. However, this does not indicate that inclusion of such a group has no place in BMS trials – for example, some regulators recommend inclusion of a 'breastfed reference group' which they consider to be a useful benchmark for the assessment of growth [6].

15a. The ethics statement in the Trial Protocol and Trial Reports clearly states how breastfeeding was supported during the trial.

Such a statement includes specific steps and information which were given to protect participants against the risk of BMS promotion within the trial.

15b. Trial methods do not involve anything that may be interpreted as an incentive to introduce BMS to an infant's diet and emphasise the superiority of breastfeeding over BMS in all literature.

Trial methods include recruitment, randomisation and treatment allocation and trial monitoring. Trials which randomise women during pregnancy or while exclusively breastfeeding, and allocate BMS to participants at this time, may be in violation of the Code. Trial literature including recruitment adverts and participant information sheets should emphasise the superiority of breastfeeding and breastmilk over BMS.

15c. Randomisation and treatment allocation do not occur until the time-point when a participant expresses an intention to introduce BMS, and participants are offered skilled breastfeeding support from a trained breastfeeding counsellor at this stage, prior to randomisation and introduction of experimental and control BMS.

Breastfeeding support should preferably come from 'independent' International Board Certified Lactation Consultants (IBCLC) who are not employed by, and have no financial relationship with, individuals or institutions with a potential commercial interest in the outcome of the trial. Use of BMS products other than experimental and control BMS should be permitted while awaiting delivery of experimental and control BMS, in order to avoid infant feeding problems.

15d. Incentives to participate in the trial do not include provision of free or discounted BMS, samples, equipment or other gifts related to BMS and its marketing. If free or discounted BMS is felt to be essential, then a similar level of reimbursement should be provided for continued breastmilk feeding.

Provision of free experimental and control BMS to trial participants is common practice in some BMS trials. If participants are already eligible for free, discounted or subsidised BMS in the trial setting through their healthcare provider or insurer, then experimental and control BMS may be provided in the same way. However, if participants would usually pay for BMS, they should not be provided with free, discounted or subsidised BMS as a consequence of their participation in the trial since this may violate the Code by acting as an incentive to introduce BMS in place of breastmilk. We acknowledge that this recommendation represents a challenge to some current BMS trial practice. If free or discounted BMS is felt to be essential then a similar level of reimbursement should be provided for continued breastfeeding or for continued use of donor breastmilk, in order to avoid providing an increased incentive for participants to introduce BMS, compared with those not participating in the trial.

15e. For trials which involve groups of infants at increased risk of a severe adverse event related to BMS use, a high level of scrutiny regarding the possibility of a negative impact on breastmilk feeding is required.

In BMS trials which include for example preterm infants, the consequences of undermining breastmilk feeding are potentially severe. Preterm infants are at increased risk of necrotising enterocolitis when BMS is used in place

of breastmilk. In a BMS trial in such high-risk infants which includes infants fed with breastmilk at enrolment or during the trial, the Trial Protocol and Trial Reports should clearly justify the use of a BMS in place of maternal or donor breastmilk for trial participants and criteria 15a, 15b, 15c, 15d should be carefully adhered to.

16. Results

These criteria for the description of trial results include description of baseline characteristics, withdrawals or losses to follow-up and presentation of trial outcomes.

16a. Trial Reports clearly describe the setting, location and timing of participant recruitment, and consider differences and similarities between the studied population and the local population where the trial was conducted.
This criterion helps readers and reviewers to better understand the precise population which was targeted in the trial, and its relationship to the population of infants for whom the BMS is intended. We recommend documenting differences between the study population and the relevant local population, including breastfeeding rates, complementary feeding and BMS use.
16b. Trial Reports include baseline demographics and clinical characteristics for each group, for all important predictors of the trial primary outcome.
Where relevant, this includes prevalence or magnitude of the trial primary outcome in each group at the time of randomisation, and the timing of BMS introduction and duration of exclusive breastfeeding in each group prior to randomisation.
16c. Trial Reports discuss any differences between experimental and control groups at baseline and consider the effect of these on any of the trial outcomes.
Statistical adjustment for these differences should be conducted, where appropriate, in order to determine the robustness of the primary analysis findings.
16d. Trial Reports include a participant flow diagram.
Participant flow diagrams detail the number of participants enrolled, number of participants randomised, number of participants in each group and number of participants analysed at each follow-up point for any of the trial outcomes.
16e. Trial Reports clearly describe any losses to follow up or withdrawal from the trial after randomisation, including reasons for the loss to follow up or withdrawal, and whether there was any relationship with the trial intervention.
Mechanisms should be put in place to reduce losses to follow up from the study as far as possible. For all participants who withdraw from the study prior to the final study visit, the principal investigator should make reasonable attempts to follow up to determine a reason for withdrawal. Information should be captured and reported concerning number of participants randomized, receiving intended intervention, completing the study protocol, and analysed for the primary and secondary outcomes in each intervention group and reasons for withdrawal or loss to follow-up from the study.
16f. Trial Reports include all data for important harms or unintended effects in each group, including whether these were thought to be a consequence of the intervention or related to BMS use.
Both number and type of adverse events in each group, and number of participants affected by at least one adverse event in each category should be reported, together with differences between groups. Any changes to the endpoints assessed or methods of assessment, compared to the protocol initially registered on the clinical trial registry, should be clearly indicated.

17. Publication

17. In the Trial Protocol the investigators acknowledge their responsibility for timely publication of all registered outcomes, and Trial Reports include all data for primary and secondary outcomes described in the Trial Protocol, including the number of participants analysed for each endpoint, in each treatment group.
The Trial Reports should include the outcome data for each primary and secondary outcome, for each intervention group and the estimated effect sizes and their precision, for the differences between groups. For example, the number in each group with an outcome, or mean and standard deviation in each group, and number analysed for that outcome in each group; then an estimated effect size for the difference between groups, and a measure of its precision. Any changes to the endpoints assessed or methods of assessment, compared to the protocol initially registered on the clinical trial registry, should be clearly indicated.

© 2020 Jarrold K et al. *JAMA Pediatrics*.

18. Conclusions

18a. Conclusions are consistent with the results, balance the benefits and harms, and consider any other relevant evidence.
Consideration of other relevant evidence includes consistency or inconsistency with other trials.
18b. Interpretations clearly distinguish clinical relevance and statistical significance.
Interpretation of trial findings should consider both statistical significance of findings and clinical relevance of effect sizes and their confidence intervals.

19. Limitations

19a. Trial Reports address trial limitations, including sources of potential bias, imprecision and, if relevant, multiplicity of analyses.
Limitations should be addressed in the Discussion section of a journal manuscript and equivalent section in other forms of Trial Report.
19b. Where appropriate, statistical methods such as multiple imputation are used to explore the effect of attrition on trial findings.
This is done in order to determine the robustness of the primary analysis findings.
19c. Trial Reports discuss the limitations of any findings which are based on analysis of participants with a minimum level of experimental or control BMS intake.
Compliance with experimental and control BMS interventions should be regularly monitored during trials, and summarised in the Trial Reports. Where appropriate, for example in trials with an equivalence or non-inferiority objective, analyses of participants with a minimum level of experimental and control BMS intake may be undertaken. Such analyses should be undertaken with an *a priori* defined minimum level of experimental and control BMS intake. This level should be stated in the Trial Protocol or in a Statistical Analysis Plan, finalised and signed prior to data lock, analysis and unblinding. Investigators should consider adjusting such analyses for potential confounders in order to determine the robustness of any findings which are not derived from ITT analysis.

20. Conflicts of Interest

20a. Sources of funding and other support for the trial are fully disclosed in the Trial Protocol and Trial Reports.
Examples of other sources of support for the trial include supply of experimental and control BMS, study materials, staff, equipment or facilities.
20b. Trial Reports declare any potential author conflict of interest.
Conflict of interest includes current, previous or anticipated receipt of funding or sponsorship from an organisation with a financial interest related to the BMS industry or the outcomes of the trial. Details of how conflict of interest statements are made and the relevant time period which they should cover are usually set by journal publisher or regulator policies.
20c. Trial Reports fully disclose any role of trial funders in the design, enrolment of participants, conduct of the trial, planning and conduct of statistical analyses, decision to publish or reporting and interpretation of the trial findings.
The role(s) of the trial funder(s) should be clearly described so that the degree of independence of trial design, conduct, analysis and reporting can be evaluated.
20d. An investigator who is independent of the BMS industry takes overall responsibility for the conduct of the trial, planning and conduct of statistical analyses, decision to publish, reporting and interpretation of the trial findings, and ensures that the planning and conduct of statistical analyses are led independently of the BMS industry.
The lead investigator of the trial should be independent of the BMS industry, or any other organisation with a financial interest related to the outcomes of the trial, and should take overall responsibility for the trial. Statistical analysis should be undertaken after the statistical analysis plan has been agreed and the database has been locked, by an independent entity in a blinded manner. We acknowledge the role that BMS industry may play in planning of statistical analyses and interpretation of findings, especially in relation to commercial aspects, but the analysis should be led independently, either by the lead investigator or by a delegated, independent entity. In general, in-house industry-led statistical planning and analysis is not appropriate, unless there is complete transparency due to audit by regulators, or full publication of all trial outcome data, such that all statistical analyses can be independently verified.

© 2020 Jarrold K et al. *JAMA Pediatrics*.

eTable 1. Essential Criteria for the Conduct and Reporting of Clinical Trials of Breastmilk Substitutes

Domain		Consensus statement
**Trial Design and Rationale**	1a	The Trial Protocol and Trial Reports include a summary of the existing evidence in the area of study and an explanation of the trial rationale
	1b	The Trial Protocol and Trial Reports include a clear description of the trial design, objectives and hypotheses
	1c	The trial design is appropriate for the trial objectives, specifying whether the trial is a superiority, non-inferiority or equivalence trial.
**Trial Registration**	2a	The Trial Protocol is registered on a clinical trial register which includes all information specified in the World Health Organization Trial Registration Data Set.
	2b	Trial Reports clearly describe any amendments to the protocol after trial commencement, and the clinical trial register is updated when significant amendments are made.
	2c	The Trial Protocol clearly defines any trial stopping rules and withdrawal criteria.
**Methods Recruitment**	3a	The Trial Protocol and Trial Reports clearly describe the pre-defined inclusion and exclusion criteria which guided participant recruitment.
	3b	The Trial Protocol and Trial Reports describe the method of participant recruitment in detail.
**BMS Composition and Formulation**	4a	The Trial Protocol and Trial Reports clearly describe the composition and formulation of the experimental and control BMS and their relationship, if any, to existing BMS products marketed anywhere in the world.
	4b	The experimental and control BMS both meet legally required compositional standards, and any instructions for safe reconstitution of BMS by trial participants are consistent with relevant national or international guidance.
	4c	The Trial Protocol and Trial Reports clearly describe any differences between experimental and control BMS which are additional to the constituent(s) of interest and consider their potential impact on the trial results.
	4d	Appropriate pre-clinical studies have been performed for previously untested components of BMS.
**Randomisation**	5a	Participants are randomly allocated to either the experimental or control group, and the procedure for randomisation is clearly described.
**Blinding**	5b	Personnel involved in enrolling participants and assigning interventions are blinded to the allocation sequence, and methods used to conceal allocation are clearly described.
	6a	The Trial Protocol and Trial Reports clearly describe methods to achieve blinding of participants' carers, or an explanation of why blinding is not feasible.
	6b	The Trial Protocol and Trial Reports clearly describe the method by which outcome assessors are kept blind to which treatment group participants were allocated throughout the trial.
**Intervention**	7a	For trials with a primary non-inferiority or equivalence objective, such as growth and tolerance trials, participants should be exclusively BMS fed at enrolment.

© 2020 Jarrold K et al. *JAMA Pediatrics*.

	7b	The Trial Protocol and Trial Reports describe how intake of experimental and control BMS is recorded during the trial, and the Trial Reports summarise experimental and control BMS intake in each treatment group during the intervention period.
	7c	Trial participants' intake of any foods other than experimental and control BMS during the intervention and data collection periods is recorded.
	7d	The age of infants at the start and end of the intervention period is appropriate for the trial objectives, and the age range at enrolment is sufficiently narrow for treatment effects to be comparable across the trial population.
**Outcome assessment**	8a	The Trial Reports clearly state the pre-defined specific primary and secondary outcomes, including timing and method of outcome assessment, and these are consistent with the clinical trial register record.
	8b	The endpoints assessed for each outcome include recognized, well-defined, measurable, interpretable, valid, reliable and clinically relevant endpoints.
	8c	For growth outcomes, trial reports should comment on whether metabolic and developmental outcomes were also evaluated.
**Surrogate endpoints**	9a	Any surrogate endpoints used to predict the outcome are appropriate, clinically relevant, measurable, interpretable and widely-accepted measurements.
	9b	If a surrogate endpoint is used, the Trial Protocol and Trial Reports discuss the rationale for using a surrogate endpoint in place of a clinical endpoint, and any uncertainty incurred.
**Adverse events**	10a	The Trial Protocol includes a valid and well recognized definition of common and anticipated adverse events.
	10b	The method for evaluating, categorising and reporting adverse events is independent of individuals or institutions with a potential commercial interest in the outcome of the trial.
**Statistical considerations**	11a	The Trial Protocol and Trial Reports include a description of how the sample size was determined and the statistical power of the trial, including expected attrition rates.
	11b	Statistical power is based on detection of an effect size for the primary outcome(s) which is both statistically significant and clinically meaningful.
	11c	Statistical methods used to compare groups for the primary and secondary outcomes are appropriate and pre-specified in the Trial Protocol.
	11d	The Trial Protocol and Trial Reports describe all pre-specified subgroup analyses and/or adjusted analyses.
**Post-hoc analyses**	12a	In the Trial Reports, any additional exploratory statistical analyses are described, acknowledged as *post hoc* analyses and justified.
	12b	Statistical analyses which were not pre-specified in the Trial Protocol are interpreted with caution and are not used as the basis for claims in the trial conclusions, or to support recommendations for infant feeding.
**Trial Ethics**	13a	The Trial Protocol and Trial Reports include a statement regarding the trial's adherence to the Declaration of Helsinki and Good Clinical Practice guidance.
	13b	Trial Reports include an International Committee of Medical Journal Editors (ICMJE) ethics statement.
**Ethics for trials in BMS-fed infants**	14	For trials where participants are all exclusively BMS fed at enrolment, such as growth and tolerance trials, carers' decision not to breastfeed should be firmly established prior to enrolment in the trial.

© 2020 Jarrold K et al. *JAMA Pediatrics*.

**Ethics for trials where some participants consume breastmilk†**	15a	The ethics statement in the Trial Protocol and Trial Reports clearly states how breastfeeding was supported during the trial.
	15b	Trial methods do not involve anything that may be interpreted as an incentive to introduce BMS to an infant's diet and emphasise the superiority of breastfeeding over BMS in all literature.
	15c	Randomisation and treatment allocation do not occur until the time-point when a participant expresses an intention to introduce BMS, and participants are offered skilled breastfeeding support from a trained breastfeeding counsellor at this stage, prior to randomisation and introduction of experimental and control BMS.
**Results**	16a	Trial Reports clearly describe the setting, location and timing of participant recruitment, and consider differences and similarities between the studied population and the local population where the trial was conducted.
	16b	Trial Reports include baseline demographics and clinical characteristics for each group, for all important predictors of the trial primary outcome.
	16c	Trial Reports discuss any differences between experimental and control groups at baseline and consider the effect of these on any of the trial outcomes.
	16d	Trial Reports include a participant flow diagram.
	16e	Trial Reports clearly describe any losses to follow up or withdrawal from the trial after randomisation, including reasons for the loss to follow up or withdrawal, and whether there was any relationship with the trial intervention.
	16f	Trial Reports include all data for important harms or unintended effects in each group, including whether these were thought to be a consequence of the intervention or related to BMS use.
**Publication**	17	In the Trial Protocol the investigators acknowledge their responsibility for timely publication of all registered outcomes, and Trial Reports include all data for primary and secondary outcomes described in the Trial Protocol, including the number of participants analysed for each endpoint, in each treatment group.
**Conclusions**	18a	Conclusions are consistent with the results, balance the benefits and harms, and consider any other relevant evidence.
	18b	Interpretations clearly distinguish clinical relevance and statistical significance.
**Limitations**	19a	Trial Reports address trial limitations, including sources of potential bias, imprecision and, if relevant, multiplicity of analyses.
	19b	Where appropriate, statistical methods such as multiple imputation are used to explore the effect of attrition on trial findings.
**Conflicts of Interest**	20a	Sources of funding and other support for the trial are fully disclosed in the Trial Protocol and Trial Reports.
	20b	Trial Reports declare any potential author conflict of interest.
	20c	Trial Reports fully disclose any role of trial funders in the design, enrolment of participants, conduct of the trial, planning and conduct of statistical analyses, decision to publish, or reporting and interpretation of the trial findings.
	20d	An investigator who is independent of the BMS industry takes overall responsibility for the conduct of the trial, planning and conduct of statistical analyses, decision to publish, reporting and interpretation of the trial findings, and ensures that the planning and conduct of statistical analyses are led independently of the BMS industry.

© 2020 Jarrold K et al. *JAMA Pediatrics*.

[†] For growth and tolerance trials, or other trials with non-inferiority or equivalence objectives, participants should be fully BMS fed and the decision not to breastfeed should be firmly established prior to enrolment in the trial. For other trials, where some participants may be receiving breastmilk at enrolment or during the intervention period, trial design and conduct should comply with the International Code of Marketing of Breastmilk Substitutes and Subsequent Relevant World Health Assembly Resolutions in order to avoid undermining breastmilk feeding.

BMS, Breastmilk Substitute.

© 2020 Jarrold K et al. *JAMA Pediatrics*.

eTable 2. Recommended Criteria for the Conduct and Reporting of Clinical Trials of Breastmilk Substitutes

Domain		Consensus statement
**Adverse events**	10c	The method for collecting information about adverse events includes a combination of checklists and open-ended questions.
**Ethics for trials where some participants consume breastmilk**†	15d	Incentives to participate in the trial do not include provision of free or discounted BMS, samples, equipment or other gifts related to BMS and its marketing. If free or discounted BMS is felt to be essential, then a similar level of reimbursement should be provided for continued breastmilk feeding.
	15e	For trials which involve groups of infants at increased risk of a severe adverse event related to BMS use, a high level of scrutiny regarding the possibility of a negative impact on breastmilk feeding is required.
**Limitations**	19c	Trial Reports discuss the limitations of any findings which are based on analysis of participants with a minimum level of experimental or control BMS intake.

† For growth and tolerance trials, or other trials with non-inferiority or equivalence objectives, participants should be fully BMS fed and the decision not to breastfeed should be firmly established prior to enrolment in the trial. For other trials, where some participants may be receiving breastmilk at enrolment or during the intervention period, trial design and conduct should comply with the International Code of Marketing of Breastmilk Substitutes and Subsequent Relevant World Health Assembly Resolutions in order to avoid undermining breastmilk feeding.

BMS, Breastmilk Substitute.

© 2020 Jarrold K et al. *JAMA Pediatrics*.

eReferences

1. Biomarkers Definitions Working G. Biomarkers and surrogate endpoints: preferred definitions and conceptual framework. *Clin Pharmacol Ther.* 2001;69(3):89-95.

2. WHO. World Health Organization. WHO Registry Criteria 2019 [Available from: https://www.who.int/ictrp/network/criteria_summary/en/].

3. Deckelbaum RJ, Adair L, Appelbaum M, et al. Infant formula: evaluating the safety of new ingredients. Washington, DC: The National Academies Press; 2004.

4. International Council for Harmonisation of Technical Requirements for Pharmaceuticals for Human Use. E6 Good Clinical Practice and E9 Statistical principles for clinical trials [Available from: https://www.ich.org/products/guidelines/efficacy/article/efficacy-guidelines.html].

5. Food and Drug Administration. Electronic Code of Federal Regulations Title 21, Chapter I, Subchapter B, Part 106, §106.96 Requirements for quality factors for infant formulas and §106.121 Quality factor assurances for infant formulas. U.S Department of Health and Human Sciences; current as of April 22, 2019. [Available from https://www.ecfr.gov].

6. Eskander M., Nguyen L. Protocol for a Growth and Tolerance Clinical Trial for Healthy Term Newborn Infants. A Guidance Document for Infant Formula Manufacturers (Draft). Bureau of Nutritional Sciences, Health Canada 5th July 2019.

7. Sedgwick P. Clinical trials: outcome measures. *BMJ.* 2015;350:h121.

8. ICH Expert Working Group. Structure and Content of Clinical Study Reports E3. ICH Harmonised Tripartite Guideline. 1995:24.

9. Schulz KF, Altman DG, Moher D, Group C. CONSORT 2010 statement: updated guidelines for reporting parallel group randomised trials. *BMJ.* 2010;340:c332.

10. European Commission. Food for infants and young children. [Available from: https://ec.europa.eu/food/safety/labelling_nutrition/special_groups_food/children_en] Accessed 9th August 2019.

11. Commission Delegated Regulation (EU) 2016/127 of 25 September 2015 supplementing Regulation (EU) No 609/2013 of the European Parliament and of the Council as regards the specific compositional and information requirements for infant formula and follow-on formula and as regards requirements on information relating to infant and young child feeding. Contract No.: EC 2016/127.

12. Food and Drug Administration. Federal Food, Drug and Cosmetic Act: Chapter IV, (1938).

13. Turck D, Bresson JL, Burlingame B, et al. Scientific and technical guidance for the preparation and presentation of an application for authorisation of an infant and/or follow-on formula manufactured from protein hydrolysates. *EFSA Journal.* 2017;15(5).

14. Food and Drug Administration. Draft Guidance for Industry: Substantiation for Structure/Function Claims Made in Infant Formula Labels and Labeling. FDA-2016-D-2241; current as of September 16, 2016. [Available from https://www.fda.gov/regulatory-information/search-fda-guidance-documents/draft-guidance-industry-substantiation-structurefunction-claims-made-infant-formula-labels-and].

15. Food and Drug Administration. Guidance for Industry: Evidence-Based Review System for the Scientific Evaluation of Health Claims. January 2009. [Available from: https://www.fda.gov/Food/GuidanceRegulation/GuidanceDocumentsRegulatoryInformation/ucm073332.htm].

16. EFSA Panel on Dietetic Products N, Allergies. Scientific Opinion on the essential composition of infant and follow-on formulae. *EFSA Journal.* 2014;12(7):3760.

17. Harman NL, Bruce IA, Callery P, et al. MOMENT–Management of Otitis Media with Effusion in Cleft Palate: protocol for a systematic review of the literature and identification of a core outcome set using a Delphi survey. *Trials.* 2013;14(1):70.

18. Association WM. World Medical Association Declaration of Helsinki. Ethical principles for medical research involving human subjects. *Bulletin of the World Health Organization.* 2001;79(4):373.

19. ICMJE. Recommendations for the Conduct, Reporting, Editing, and Publication of Scholarly work in Medical Journals 2018 [updated December 2018]. [Available from: http://www.icmje.org/recommendations].

© 2020 Jarrold K et al. *JAMA Pediatrics*.

20. Sterne JAC, Savović J, Page MJ, et al. RoB 2: a revised tool for assessing risk of bias in randomised trials. BMJ (in press).

21. World Health Organization. International Code of Marketing of Breastmilk Substitutes. Geneva: World Health Organization; 1981.

22. Codex Alimentarius Commission. Standard for infant formula and formulas for special medical purposes intended for infants CODEX STAN 72-1981. Revised in 2007 and last amended in 2016. [Available from: http://www.fao.org/fao-who-codexalimentarius/codex-texts/list-standards/en/].

23. Leonardi-Bee J, Garcia-Larssen V, Jarrold K, Mundell A, Man A, Ducharme-Smith K. Systematic review of the methodological quality of clinical trials of breast milk substitutes: PROSPERO CRD42018091928 2018 [Available from: http://www.crd.york.ac.uk/PROSPERO/display_record.php?ID=CRD42018091928].

24. WHO. World Health Organization. The WHO International Clinical Trials Registry Platform (ICTRP) 2019 [Available from: http://www.who.int/ictrp/network/trds/en/].

25. WHO. World Health Organization. WHO Primary Registries 2019 [Available from: http://www.who.int/ictrp/network/primary/en/].

26. Committee ES, Hardy A, Benford D, et al. Guidance on the risk assessment of substances present in food intended for infants below 16 weeks of age. *EFSA Journal.* 2017;15(5):e04849.

27. Food and Drug Administration. Generally Recognized as Safe (GRAS). September 2018. [Available from: https://www.fda.gov/food/food-ingredients-packaging/generally-recognized-safe-gras].

28. WHO. World Health Organization Multicentre Growth Reference Study Group. WHO Child Growth Standards: Length/height-for-age, weight-for-age, weight-for-length, weight-for-height and body mass index-for-age: Methods and development. Geneva: World Health Organization, 2006. [Available from: https://www.who.int/childgrowth/standards/technical_report/en/].

29. Radlowski EC, Johnson RW. Perinatal iron deficiency and neurocognitive development. *Frontiers in human neuroscience.* 2013;7:585.

30. Dunning AJ. A model for immunological correlates of protection. *Statistics in medicine.* 2006;25(9):1485-1497.

31. Fleming TR, Powers JH. Biomarkers and surrogate endpoints in clinical trials. *Statistics in medicine.* 2012;31(25):2973-2984.

© 2020 Jarrold K et al. *JAMA Pediatrics*.
